# A national cross-sectional study on the influencing factors of low HPV vaccination coverage in mainland China

**DOI:** 10.3389/fpubh.2022.1064802

**Published:** 2023-01-16

**Authors:** Xiangju Yin, Mengrui Zhang, Fei Wang, Yue Huang, Yuyao Niu, Pu Ge, Wenli Yu, Yibo Wu

**Affiliations:** ^1^School of Emergency Management, Henan Polytechnic University, Jiaozuo, China; ^2^School of Resource and Environment, Henan Polytechnic University, Jiaozuo, China; ^3^State Key Laboratory of Cognitive Neuroscience and Learning, Beijing Normal University, Beijing, China; ^4^Department of English, Faculty of Arts and Humanities, University of Macau, Macau, China; ^5^School of Foreign Languages, Weifang University of Science and Technology, Weifang, China; ^6^School of Public Health, Peking University, Beijing, China

**Keywords:** self-efficacy, family health literacy, restricted cubic spline, cervical cancer, HPV vaccination

## Abstract

**Background:**

HPV vaccine can block the infection of high-risk human papillomavirus and is an important measure to effectively reduce the incidence of cervical cancer and precancerous lesions. However, the HPV vaccination rate is still low in China. There are many factors. Therefore, it is important to study the influencing factors to provide basis for promoting the formulation of vaccination strategies.

**Methods:**

This study used a multi-stage sampling method to conduct a face-to-face questionnaire survey on women in different regions of China. The new general self-efficacy scale was used to measure the self-efficacy of the respondents. The short form of family health scale measured their family health. The *t*-test and binary Logistic regression analysis were used to screen the influencing factors of HPV vaccination. Restricted cubic spline model was used to analyze the influence trend of self-efficacy and family health on HPV vaccination rate.

**Results:**

(1) The HPV vaccination rate was low, especially in the ≤18 group. The place of residence, capita household income/month, individual self-efficacy and family health had a significant impact on HPV vaccination. (2) The restricted cubic spline model showed that self-efficacy positively promoted HPV vaccination, the correlation strength was statistically significant (χ^2^ =27.64, *P*<0.001) and non-linear (χ^2^ = 12.49, *P* = 0.0004); The poor family health hindered HPV vaccination, and the association strength was statistically significant (χ^2^ = 47.81, *P* < 0.001) and non-linear (χ^2^ = 9.96, *P* = 0.0016).

**Conclusion:**

It is necessary to strengthen the health education of HPV vaccination knowledge in the population to eliminate the hesitancy of vaccination. Free HPV vaccination strategies should be developed and encourage people of appropriate age to receive as early as possible. Self-efficacy and family health should be enhanced to increase HPV vaccination rate, so as to achieve the goal of reducing the incidence of cervical cancer and protecting women's health.

## Introduction

Cervical cancer seriously endangers women's health. Worldwide, it ranks the fourth most frequent cancer in women. There were estimated 604,000 new cases and 342,000 deaths from cervical cancer in 2020 (1). In China, cervical cancer incidence rate and mortality rate ranked the second among female tumors, and there were 110,000 new cases and 59,060 deaths of cervical cancer in 2020 (2). Persistent human papilloma virus (HPV) infection is a major risk factor for cervical epithelial carcinogenesis. It is estimated that HPV-attributable cancer cases and age-standardized incidence rate will reach 214,077/100,000 and 9.35/100,000 respectively by 2030, of which 87.7% are cervical cancer in China (3). Cervical cancer has brought heavy economic and social burden.

The developed HPV vaccine can block the persistent infection of high-risk HPV, thereby effectively reduce the incidence of cervical cancer and precancerous lesions. The promotion of HPV vaccination among the appropriate age groups is an effective measure to prevent cervical cancer (4). Compared with those women who were not vaccinated, women who received HPV vaccine before the age of 17 had an 88% lower risk of cervical cancer, while women who received the vaccine between the ages of 17 and 30 had a half lower risk of cervical cancer. HPV vaccine can effectively inhibit the incidence of cervical cancer, and the earlier the vaccination, the better effect (5). The United States Centers for Disease Control and Prevention (CDC) predicted that sexual activity without HPV vaccine would be a factor of up to 80.0% HPV infection (6).A meta-analysis of more than 60 million people in 14 countries over an 8-year period shows that countries with multiage vaccination and high vaccination coverage had greater group effect of vaccine protection (7).

However, the current situation of HPV vaccination in many countries is not optimistic. Global HPV immunization coverage was estimated at 12.2% in 2018 (8). Global coverage for the HPV final dose was estimated at 15% in 2019 (9). HPV vaccination rates is also low in China, A report in 2021 pointed out that the coverage rate of hpv vaccine for adolescents was lower than 3%, and that for the whole population was lower than 6% (10). However, the awareness of HPV vaccine among reproductive women in some areas of China in 2018 showed that 53.8% of them knew the HPV vaccine (11). There is a significant gap between the awareness rate and the vaccination rate, suggesting that there may be a variety of factors affecting the appropriate population to vaccinate HPV vaccine. Therefore, in order to further analyze the reasons, we selected some cities and regions in China to carry out a cross-sectional survey and study on HPV vaccination. It is of great social significance to promote and improve the HPV vaccination rate and reduce the incidence of cervical cancer.

## Materials and methods

### Study design

In this study, from July to September 2021, we selected 2–6 cities from each province and autonomous region in Chinese Mainland by random number table method, and 120 cities were included. Based on the results of the “2021 Seventh National Population Census”, a sample survey was conducted by gender, age, urban and rural distribution and other quota attributes. Meanwhile, we collected investigators from all over the country in China and conducted unified training for the investigators before issuing the questionnaires. The investigators distributed the questionnaire to the public in their respective areas through the link of the questionnaire star platform “WJX” (Ranxing Information Technology Co., Ltd., Changsha, Hunan, China). Before the survey, the subjects were informed and agreed, and then invited to click the questionnaire link to answer. A total of 5,994 women questionnaires were collected and we screened 5,959 valid questionnaires, with the 99.4% effective rate. 4,340 cases (72.8%) lived in urban areas, 1,619 cases (27.2%) lived in rural areas. There were 1,896 cases (31.8%) with capita household income/month ≤3,000 yuan, 2,303 cases (38.6%) with 3,001–6,000 yuan, 962 cases (16.1%) with 6,001–9,000 yuan, and 798 cases (13.4%) with ≥9,001 yuan. 623 cases (10.5 %) were ≤ 18 years old, 3,006 cases (50.4 %) were 19–40 years old, 1,906 cases (32.0 %) were 41–65 years old, and 424 cases (7.1 %) were ≥65 years old. The marital status was 2,427 (40.7%) unmarried and 3,532 (59.3%) married. There were 1,378 cases (23.1%) with education level of junior high school and below, 988 cases (16.6%) with secondary and high school, 759 cases (12.7%) with college, and 2,834 cases (47.6%) with a university degree and above. The medical expenses of 4,651 cases (78.1%) were paid by medical insurance or public funds, while 1,308 cases (21.9%) were self-paid.

Inclusion criteria: ① Respondents read the informed consent form and participate in this study voluntarily. ② They are able to complete the online questionnaire or with the help of investigators. ③ Respondents have basic cognitive ability and understand the meaning of each item in the questionnaire. Exclusion criteria: ① Questionnaires with unqualified addresses. ② Questionnaires from male respondents. ③ Questionnaires with conflicting information.

### Instruments

To ensure the scientificity and effectiveness of the questionnaire, it is divided into two parts: basic information (including basic family information and basic personal information) and standard scales (including Perceived Social Support Scale, New General Self-Efficacy Scale, Family Health Scale, Health Literacy Scale).

Perceived Social Support Scale (PSSS) (12). The scale was compiled by Zimet et al. to access people's perception of social support. It is a social support scale that emphasizes individual self-understanding and self-feeling. Individuals' perceived levels of support from various social support sources, such as family, friends, and others, were measured separately. The total score was used to reflect the individual's overall level of social support. The Cronbach's alpha of the PSSS was 0.952. The scale has a total of 12 items and consists of three dimensions, each of which contains 4 items. Each item is scored on a 7-point Likert scale, ranging from strongly disagree (0 points) to strongly agree (6 points). The scale's total score ranges from 0 to 72, with higher scores indicating higher perceptions of social support.

New General Self-Efficacy Scale (NGSES) (13). The scale was developed after the revision of Chen G's general self-efficacy scale (14). Bandura (15) firstly defined self-efficacy as people's expectations, perceptions, confidence, or beliefs about the ability to successfully implement the action process required to achieve a specific goal. The Cronbach's alpha of the NGSES was 0.940. This scale consists of 8 items, each of which is scored on a 5-point Likert scale, ranging from strongly disagree (1 point) to strongly agree (5 points), with a total score range of 8–40, with higher scores Indicates that the subjects have a higher level of self-efficacy.

Health Literacy Scale, a 12-item short-form HL questionnaire (HLS-SF12) (16). Health literacy (HL) aims to assist people in making health-related decisions and taking appropriate actions to manage their health. This concept was proposed by Sørensen in 2012 (17). The Cronbach's alpha of the HLS-SF12 is 0.937. Each health-related task's perceived difficulty was rated on a 4-point Likert scale from very difficult (1 point) to very easy (4 point), with a total score range of 4–48, with higher scores indicating higher health literacy.

A Short Form of the Family Health Scale (FHS-SF), The FHS-SF contains four dimensions: family social and emotional health processes, family healthy lifestyle, family health resources, and family external social supports (18). The Cronbach's alpha of the FHS-SF was 0.846. The FHS-SF is a 10-item scale with a 5-point Likert scale ranging from strongly disagree (1 point) to strongly agree (5 point), with a total score ranging from 5 to 50, with higher scores indicating better family health.

### Statistical methods

In this study, SPSS25.0 was used for statistical analysis of the data, t and χ^2^ tests were used to compare the rate and mean between the two groups, and the scale scores were expressed as mean ± standard deviation ($\bar{x}$ ± *s*). Binary logistic regression was used for multivariate analysis. Restricted cubic spline model was used to analyze the influence trend of self-efficacy scale and family health on HPV vaccination rate. All tests were two-sided, and the significance level was set at 0.05.

### Quality control

Two rounds of pre investigation and two rounds of expert consultation were completed before the formal investigation. Trained investigators handed out questionnaires to investigators face to face and register codes. We immediately summarized, evaluated and fed back the collected questionnaires. The logic check and data filtering were performed back-to-back by two people. Once singular values were found in the data, it was necessary to find the original questionnaire and check with the investigators to ensure the reliability and authenticity of the data.

## Results and analysis

### Comparison of differences between HPV vaccinated and unvaccinated groups

The survey found that the number of unvaccinated women was greater than that of vaccinated women (Table 1). There were significant differences between the HPV vaccinated group and the unvaccinated group in place of residence, ages, highest education levels, capita household income/month, self-efficacy, family health and health literacy and has statistical significance (*P* < 0.05), But it did not show marked differences in marital status, type of medical insurance and the perceive social support (*P* > 0.05).

**Table 1:** Comparison of differences between HPV vaccinated and unvaccinated groups.

**Indicators**	**Group**	**Non HPV vaccinated group**	**Non HPV vaccinated group**	**χ^2^ or t**	***P***
Place of residence	Urban	3,309 (76.24%)	1,031 (23.76%)	32.176	<0.001
	Rural	1,345 (83.08%)	274 (16.92%)		
Age (years)	≤ 18	546 (87.64%)	77 (12.36%)	112.734	<0.001
	19–40	2,192 (72.92%)	814 (27.08%)		
	41–65	1,544 (81.01%)	362 (18.99%)		
	≥ 66	372 (87.74%)	52 (12.26%)		
Highest education levels	Junior high school and below	1,177 (85.41%)	201 (14.59%)	68.495	<0.001
	Secondary and High School	790 (79.96%)	198 (20.04%)		
	College	562 (74.04%)	197 (25.96%)		
	Undergraduate	2,125 (74.98%)	709 (25.02%)		
Marital Status	Unmarried	1,924 (79.27%)	503 (20.73%)	3.302	0.069
	Married	2,730 (77.29%)	802 (22.71%)		
Type of medical insurance	Medical insurance or public funding	3,625 (77.94%)	1,026 (22.06%)	0.318	0.573
	Self-financed	1,029 (78.67%)	279 (21.33%)		
Capita household income/month (yuan)	≤ 3,000	1,579 (83.28%)	317 (16.72%)	74.23	<0.001
	3,001–6,000	1,806 (78.42%)	497 (21.58%)		
	6,001–9,000	717 (74.53%)	245 (25.47%)		
	≥9,001	552 (69.17%)	246 (30.83%)		
Scale	NGSES	28.69 ± 5.061	29.06 ± 5.631	−2.162	0.031
	PSSS	61 ± 12.284	61.07 ± 13.342	−0.152	0.879
	FHS-SF	38.58 ± 6.420	37.86 ± 6.763	3.432	0.001
	HLS-SF12	36.67 ± 5.722	37.28 ± 6.264	−3.156	0.002

### Analysis of influencing factors of HPV vaccination by binary logistic regression

Based on the results of t and χ^2^ test, the significant factors were further analyzed by binary Logistic regression. Age, Place of residence, Capita household income/month, highest education levelsl, the HLS-SF12, the FHS-SF, and the NGSES were used as independent variables, and the HPV vaccination status was used as the dependent variable. The assigned values are shown in Table 2.

**Table 2:** Variables and dummy variables assignment table.

**Variable name**	**Assignment description**
HPV vaccination	No = 0, Yes = 1
Place of residence	Rural = 0, Urban = 1
Age	≤18 = 0, 19–40 = 1, 41–65 = 2, ≥66 = 3
Capita household income/month (yuan)	≤ 3,000 = 0, 3,001–6,000 = 1, 6,001−9,000 = 2, ≥9,001= 3
Highest education level	Junior high school and below = 0, junior college and high school = 1, college = 2, university undergraduate and above = 3
HLS-SF12, FHS-SF, NGSES	Continuous variables

The binary logistic regression equation was constructed by incorporating place of residence, highest education level, multiple scales, etc. The results show that Table 3: the influence of place of residence on HPV vaccination is statistically significant (OR = 1.286, 95%CI 1.097–1.508, *P* = 0.002). The influence of age on HPV vaccination is statistically significant (*P* < 0.001). The influence of capita household income/month on HPV vaccination is statistically significant (*P* < 0.001). Family health had a negative impact on HPV vaccination (OR = 0.968, 95%CI 0.956–0.979, *P* < 0.001). The influence of self-efficacy on HPV vaccination is statistically significant (OR = 1.026, 95%CI 1.011–1.041, P= 0.001).

**Table 3:** Multifactorial logistic regression analysis of the effect of HPV vaccination.

**Variables**	**Beta value**	**S.E**.	**Wald value**	***P*-value**	**OR(95%CI) value**
Urban	0.252	0.081	9.583	0.002	1.286 (1.097–1.508)
Age			66.785	<0.001	
19–40	0.904	0.134	45.554	<0.001	2.470 (1.900-3.212)
41–65	0.455	0.138	10.914	0.001	1.557 (1.204-2.066)
≥66	0.194	0.200	0.941	0.332	1.214 (0.821–1.796)
Capita household income/month (yuan)			47.047	<0.001	
3,001–6,000	0.232	0.83	7.783	0.005	1.261 (1.071–1.484)
6,001–9,000	0.443	0.101	19.046	<0.001	1.557 (1.276–1.900)
≥9,001	0.694	0.106	42.914	<0.001	2.002 (1.626-2.463)
Highest education level			50,842	0.12	
Secondary and High School	0.169	0.117	2.094	0.148	1.184 (0.942–1.489)
College	0.277	0.122	5.139	0.023	1.319 (1.038–1.676)
Undergraduate and above	0.120	0.106	1.263	0.261	1.127 (0.915–1.388)
HLS-SF12	0.005	0.006	0.714	0.398	1.005 (0.993–1.018)
FHS-SF	−0.033	0.006	31.056	<0.001	0.968 (0.956–0.979)
NGSES	0.026	0.008	11.501	0.001	1.026 (1.011–1.041)
Constants	−2.187	0.285	58.716	<0.001	0.112

### The influence trend of self-efficacy and family health on HPV vaccination

#### Trends in the influence of self-efficacy on HPV vaccination

**Figure 1:**
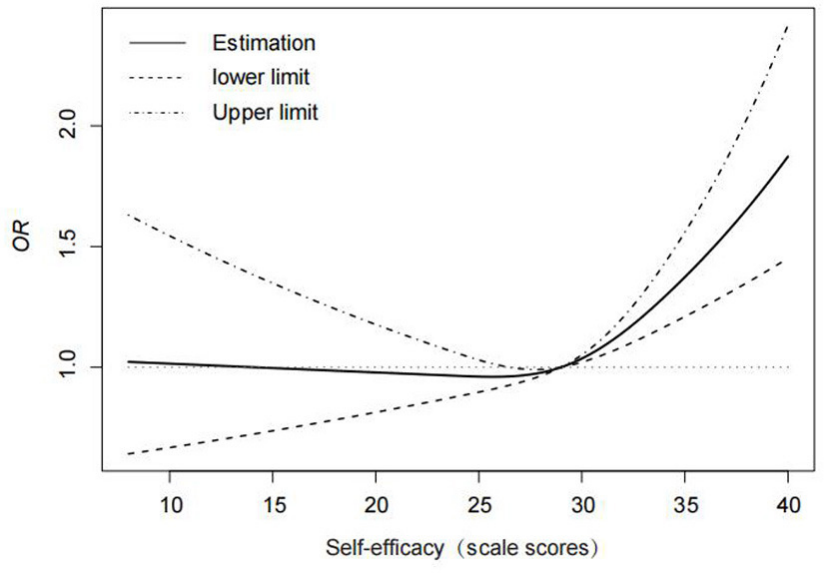
Trend of the effect of self-efficacy on HPV vaccination.

A restricted cubic spline model was used to analyze the trend of self-efficacy and vaccine influence on HPV vaccination, and three nodes (P25, P50, and P75) were selected based on NGSES scores, with the value of the reference point (at HR/OR = 1) being the median P50 and the OR (95% CI) of the P25 and P75 nodes: 0.974 (0.892–1.065), respectively 1.1703 (1.103–1.241). After controlling for confounders such as age, household income, literacy, and family health scale, the results revealed that the association between self-efficacy and HPV vaccination was statistically significant (χ^2^ = 27.64, *P* < 0.001) in a non-linear manner (χ^2^ = 12.49, *P* = 0.0004). The effect on HPV vaccination was not significant when self-efficacy was below the value of 29 of P50, and when it reached above the value of 29 of P50, the effect on HPV vaccination was significantly enhanced with an increase in self-efficacy, which had a facilitative effect (Figure 1).

#### Trends in the influence of family health on vaccination

**Figure 2:**
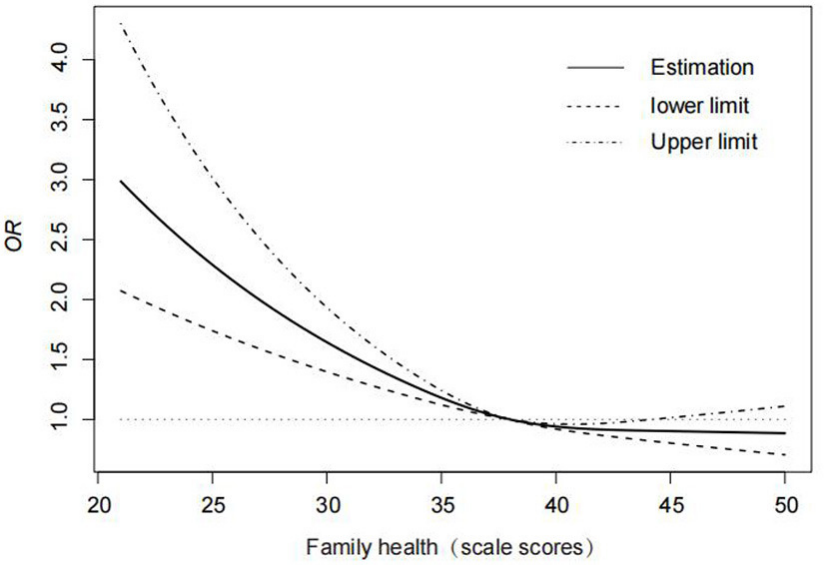
Trends in the influence of family health on vaccination.

To analyze the trend of the effect of the family health scale on HPV vaccination, a restricted cubic spline model was used, and three nodes (P25, P50, and P75) were selected based on the scores of the FHS-SF, with the value of the reference point (at HR/OR = 1) being the median P50 and the OR (95% CI) of the P25 and P75 nodes: 1.264 (1.174–1.360), respectively 0.906 (0.823–0.998). After controlling for confounders such as age, household income, literacy, and self-efficacy, the results revealed that the association between the FHS-SF and vaccination was statistically significant (χ^2^ = 47.81, *P* < 0.001) in a non-linear fashion (χ^2^ = 9.96, *P* = 0.0016). When the FHS-SF value was lower than P50 (38), the hindering effect on HPV vaccination was significant, and the negative effect weakened or disappeared as the family health level increased (Figure 2).

## Discussion

According to World Health Organization data, high-risk HPV infection is linked to 99% of cervical cancer cases. HPV vaccination is the most effective method to prevent HPV infection, and it is a primary preventive measure to prevent and control HPV infection-related diseases. The results of this study show that the vaccination rate of HPV vaccine is significantly low, only 24.39%. Chinese Mainland introduced GSK 2vHPV vaccine in August 2016, 4vHPV vaccine and 9vHPV vaccine were introduced in 2017 and 2018, respectively. The HPV vaccine has been introduced into China for a relatively short time, which may be one of the reasons why HPV vaccination is low. The study found that the place of residence, capita household income/month, individual self-efficacy and family health had a significant impact on HPV vaccination, but marital status, type of medical insurance, highest education levels, and perceived social support scale had little effect.

We found that the vaccination rate of women aged ≤18 is only 12.36%, which may be related to the low awareness and acceptance of HPV vaccine by parents. A Meta analysis of the awareness and acceptance of HPV vaccine among parents of teenagers in Chinese Mainland shows that the awareness rate of HPV and HPV vaccine among parents in Chinese Mainland is 28.21 and 18.91%, respectively (19). Some parents of teenagers mistakenly think that HPV vaccine will affect their development, and even some parents think that their children will not be infected with HPV when they are young. It is of little significance to vaccinate children with HPV vaccine, so they are unwilling to vaccinate their children. Therefore, the best time for vaccination is taken (20). Improving parents' awareness can improve the vaccination rate of teenagers. In addition, research shows that parents' acceptance of HPV vaccine for girls is related to the price of HPV vaccine. Parental acceptability of HPV vaccination were 27.4% at market price to about 50% if the price was halved and to 60% if free HPV vaccines were available (21). Incorporating HPV vaccine into the national immunization program can improve the coverage rate of HPV vaccine for adolescents. The HPV vaccination rate of the 19–40 year old group is relatively high in this study, probably because they have financial resources, and young adult women are at the better age to receive HPV vaccine, because they have no chance to receive HPV vaccine in adolescence. In addition, this group is at high risk of cervical cancer (22, 23). Therefore, they should be vaccinated against cervical cancer and screened regularly. In this survey, women over 45 years old and over 66 years old are included. Obviously, their vaccination rate is very low, because vaccination for the elderly can only be effective for the susceptible population (that is, the uninfected population exposed to new infections, but the probability of new exposure decreases with age). In addition, the elderly who are vaccinated with HPV vaccine are not cost-effective (24). So the vaccination rate should be strengthened in the appropriate age group.

Women whose permanent residence is in urban areas have a significantly higher HPV vaccination rate than rural women. It may be because rural women have weak awareness of personal hygiene and health examination, and rural medical facilities and conditions are relatively poor compared to urban areas. A study by Joseph Rujumba found similar results, with barriers such as low individual knowledge of the vaccine, shortage of health workers and poor sanitation facilities, and limited community participation interacting to make routine HPV vaccination difficult for rural women (25).

Women with higher capita household income/month were more likely to receive the HPV vaccine. It may be because women with high income are consciously concerned about the HPV vaccine. Because HPV vaccines are expensive and “one shot is hard to get”, women with lower incomes do not have the ability and energy to afford HPV vaccines. The study of Emma Altobelli showed that European countries with higher incomes had higher screening and immunization coverage. Compared with countries with lower middle incomes, these screening and immunization reduced the incidence and mortality of cervical cancer (26). This is consistent with the results obtained in our survey, in that higher income groups are in a position to be screened and have the ability to be vaccinated against HPV. Thus they will reduce morbidity and mortality of cervical cancer.

In this study, a restricted cubic spline model was used to further analyze the impact of the NGSES and FHS-SF on HPV vaccination trends. The restricted cubic spline model combines the spline function with the generalized linear model (linear regression, logistic regression, cox regression). Which overcomes the defects of regression and shows the influence of nonlinear relationship more intuitively (27). The spline curve is essentially a piecewise polynomial function, which is limited by certain control nodes. The nodes are placed in multiple positions within the data range. The type of polynomial and the number and position of the nodes determine the type of spline curve. In most cases, the position of the nodes has little effect on the fitting of the restricted cubic spline, unless the distribution of the node positions is extremely uneven, and the number of nodes is relatively a more critical parameter.Three nodes were selected in this study, namely P25, P50, and P75. Because when the number of nodes is 3, the AIC value of the model (Akaike Information Criterion) is the smallest, indicates the fitting of the model is optimal.

Self-efficacy affects people's choices and behaviors, and HPV vaccination intention is positively correlated with self-efficacy (28). Schaefer Ziemer study also showed that women who received HPV vaccine had higher self-efficacy (29). This study applied restricted cubic spline model analysis and found that there was a nonlinear association between self-efficacy and HPV vaccination after adjusting for confounding factors such as age, family income, education level, and family health. When the score of NGSES was below 29, the curve changed slowly, and the OR value was close to 1, indicating that NGSES score within P50 has little effect on the improvement of HPV vaccination rate. However, when the score of NGSES reached P50 or above, the curve showed a sharp upward trend, indicating that the self-efficacy reached a certain degree. With the increase of self-efficacy, the HPV vaccination rate also increased significantly. This study confirms the relationship between self-efficacy as an independent factor and vaccination rates, and the improvement of self-efficacy is a suitable intervention target for HPV vaccination (30). Through strengthening the education and publicity of the safety and effectiveness of vaccines, the self-efficacy of residents can be improved to a greater extent, the barriers to vaccination can be reduced, and the vaccination rate can be increased (31).

Family health is a resource of the family unit that develops from the intersection of the health, interactions of each family member, and the family's social, emotional, economic, medical resources. This study confirmed that family health affects the HPV vaccination of family members. After adjusting the confounding factors, the restricted cubic spline model was used to analyze the relationship between the family health scale score and the HPV vaccine coverage, which showed a nonlinear association.When the score is <38, the curve is declining, indicating that when the family health scale score is <P50, the vaccination rate is significantly affected, and poor family health is an obstacle to HPV vaccination. Once P50 was reached, the change in HPV vaccination rates leveled off as the family Health Scale score increased. It may be that when the family health scale score reaches the median above, the negative influence of family health status on HPV vaccination was relieved. Low awareness and acceptance of HPV vaccine among parents in the family will prevent their children from receiving HPV vaccine (20, 21). People with higher income levels have higher household health scores (32). Family members can be helped to overcome barriers to HPV vaccination (such as paying a high fee for the vaccine) (33). The family not only creates the family environment and provides family members with access to personal health resources. Moreover, the collective health of the family unit is a strong predictor of individual health. The health behaviors of family members can have certain mutual influence (34). Parental health behaviors and family support for HPV vaccination are associated with increasing vaccination intention in young adults, which are potential targets for interventions to promote HPV vaccination (35, 36). In the health care HPV vaccination plan, family health data is an important source of information. Therefore, it is necessary to provide services or health education interventions to improve family health.

In order to reduce the incidence rate and mortality of cervical cancer, the World Health Organization (WHO) announced the global strategic goal of 2030, advocating that the vaccination rate of 15 year old girls against HPV should reach more than 90% (37). However, we are still far from this goal. Therefore, policy guidance should be strengthened according to the influencing factors. Influenced by the price of vaccine, some people cannot be vaccinated. Free vaccination can be gradually promoted, and finally HPV vaccine will be included in the immunization plan, so that appropriate age women can be vaccinated as soon as possible. At present, some cities in China have carried out free vaccination programs for girls aged 13–15 in junior middle schools. At the same time, health education should be strengthened through medical institutions, schools and communities, including the most concerned about vaccine safety, effectiveness, optimal vaccination time, impact on children's development, etc. The network platform can also be used for propaganda, to improve the family health and self-efficacy of the masses, and to increase the acceptance of HPV vaccine and vaccination awareness.

### Limitations

Men who have sex with men (MSM) are at a high risk of infecting HPV (38). Men should be vaccinated to protect themselves and their future sexual partners. Only females were investigated in this study, so males will be included in subsequent studies and expand the sample size. In addition, in the study, although we investigated and found out some influencing factors of HPV vaccination, However, there are still unknown confounding factors, which need to be further explored to find solutions.

## Conclusion

In this cross-sectional survey, we found that the HPV vaccination rate in Chinese women was still relatively low, There is an urgent need to increase the vaccination rate in the appropriate age women, especially in the optimal age population for HPV vaccination. In addition to the differences between rural and urban residential areas, the family monthly income gap have a significant influence on the HPV vaccination rate, and self-efficacy and family health also have an effect. It is necessary to strengthen the scientific publicity of HPV vaccine in rural areas and people with low education level to improve the awareness of HPV vaccine. Take the enhancement of self-efficacy and family health as the target to improve the vaccination rate, eliminate people's worries and hesitations about HPV vaccination, and improve the awareness of disease prevention. The state actively promotes the free vaccination program for young people. At the same time, the government and health departments continue to improve vaccination regulatory policies to ensure vaccine safety. More people can be effectively protected by vaccination and the incidence of cervical cancer can be reduced.

## Data availability statement

The original contributions presented in the study are included in the article/supplementary material, further inquiries can be directed to the corresponding author.

## Ethics statement

The study was conducted in accordance with the Declaration of Helsinki, and approved by the Ethics Committee of Jinnan University (JUNKY-2021-018, 2021.08.26). Written informed consent to participate in this study was provided by the participants' legal guardian/next of kin.

## Author contributions

Writing-original draft, review, and editing: XY. Project administration and supervision: YW. Investigation: YN, PG, and WY. Methodology: FW. Software and writing—original draft: MZ and YH. All authors contributed to the article and approved the submitted version.

## References

[1] Sung H Ferlay J Siegel RL Laversanne M Soerjomataram I Jemal A. Global Cancer statistics 2020: GLOBOCAN estimates of incidence and mortality worldwide for 36 cancers in 185 countries. CA Cancer J Clin. (2021) 71:209–49. 10.3322/caac.21660 33538338

[2] Huang Y Chen C Wang L Wu H Chen T Zhang L. HPV Vaccine hesitancy and influencing factors among university students in China: a cross-sectional survey based on the 3cs model. Int J Environ Res Public Health. (2022) 19:14025. 10.3390/ijerph192114025 36360905 PMC9657119

[3] Duan R Xu K Huang L Yuan M Wang H Qiao Y. Temporal trends and projection of cancer attributable to human papillomavirus infection in China, 2007-2030. Cancer Epidemiol Biomarkers Prev. (2022) 31:1130–6. 10.1158/1055-9965.EPI-21-1124 35266990

[4] The National Cancer Institute. Large Study Confirms that HPV Vaccine Prevents Cervical Cancer. Available online at: https://www.cancer.gov/news-events/cancer-currents-blog/2020/hpv-vaccine-prevents-cervical-cancer-sweden-study (accessed on October 14, 2020).

[5] Lei J Ploner A Elfström KM Wang J Roth A Fang F. HPV vaccination and the risk of invasive cervical cancer. New Engl J Med. (2020) 383:1340–8. 10.1056/NEJMoa1917338 32997908

[6] CDC. Reasons to Get Vaccinated. United States: Center for Disease Control and Prevention. (2019). Available online at: https://www.cdc.gov/hpv/parents/vaccine/six-reasons.html (accessed on March 26, 2019).

[7] Drolet M Bénard É Pérez N Brisson M; HPV Vaccination Impact Study Group. HPV Vaccination Impact Study Group. Population-level Impact and Herd Effects Following the Introduction of Human Papillomavirus Vaccination Programmes: Updated Systematic Review and Meta-analysis. Lancet. (2019) 394:497–509. 10.1016/S0140-6736(19)30298-3 31255301 PMC7316527

[8] Spayne J Hesketh T. Estimate of global human papillomavirus vaccination coverage: analysis of country-level indicators. BMJ Open. (2021) 11:e052016. 10.1136/bmjopen-2021-052016 34475188 PMC8413939

[9] Bruni L Saura-Lázaro A Montoliu A Brotons M Alemany L Diallo MS. HPV Vaccination Introduction Worldwide and WHO and UNICEF estimates of national HPV immunization coverage 2010–2019. Prev Med. (2021) 144:106399. 10.1016/j.ypmed.2020.106399 33388322

[10] Cervical Cancer Prevention Committee of Guangdong. Preventive Medicine Association, Guangdong Expert Consensus on HPV Vaccine Application to Eliminate Cervical Cancer. J Chin Phys. (2021) 23:1303–15. 10.3760/cma.j.cn431274-20210819-00911

[11] Zhang XS Di JL Wang XY Landais E Pantophlet R. Analysis on awareness of human papillomavirus vaccine among reproductive women in part of China. Chin J Woman Child Health Res. (2022) 33:89–93. 10.3969/j.issn.1673-5293.2022.11.015

[12] Cohen S Matthews KA. Social support, type a behavior, and coronary artery disease. Psychosom Med. (1987) 49:325–30. 10.1097/00006842-198707000-00001 3615761

[13] Chen G Gully SM Eden D. Validation of a New General Self-Efficacy Scale. Organ Res Methods. (2001) 4:62–83. 10.1177/109442810141004

[14] Feng X Chen XY. Realizability and validity of new general self-efficacy scale (NGSSE). J Mudanjiang Normal Univ. (2012) 4:127–9. 10.13815/j.cnki.jmtc(pss).2012.04.042

[15] Bandura A. Self-efficacy: toward a unifying theory of behavioral change. Psychol Rev. (1977) 84:191–215. 10.1037//0033-295x.84.2.191 847061

[16] Duong TV Aringazina A Kayupova G Nurjanah Pham TV Pham KM. Development and validation of a new short-form health literacy instrument (HLS-SF12) for the general public in six Asian countries. Health Lit Res Pract. (2019) 3:91–102. 10.3928/24748307-20190225-01 31294310 PMC6607763

[17] Sorensen K Van den Broucke S Fullam J Doyle G Pelikan J Slonska ZC. Health literacy and public health: a systematic review and integration of definitions and models. BMC Pub Health. (2012) 12:80. 10.1186/1471-2458-12-80 22276600 PMC3292515

[18] Wang F Wu Y Sun X Wang D Ming WK Sun X. Reliability and validity of the Chinese version of a short form of the family health scale. BMC Prim Care. (2022) 23:108. 10.1186/s12875-022-01702-1 35524178 PMC9077878

[19] Shi JJ Zhang XX Zheng H Yu WZ. Awareness and Acceptance of Human Papillomavirus Vaccine Among Parents of Adolescents in Chinese Mainland: a Meta-analysis. Chin J Vaccine Immunization. (2019) 25:464–70. 10.19914/j.cjvi.2019.04.023

[20] Ke H. Wang Lp, Deng Ll, Liu LJ, Li CP. Study on the Problems and Countermeasures in the Promotion of Human Papillomavirus Vaccination. Jiangsu J Prevent Med. (2021) 32:428–30. 10.13668/j.issn.1006-9070.2021.04.013

[21] Wang Z Wang J Fang Y Gross DL Wong MCS Wong ELY. Parental acceptability of HPV vaccination for boys and girls aged 9–13 years in China—A population-based study. Vaccine. (2018) 36:2657–65. 10.1016/j.vaccine.2018.03.057 29606519

[22] Zhou HL Zhang W Zhang CJ Wang SM Duan YC Wang JX. Prevalence and distribution of human papillomavirus genotypes in Chinese women between 1991 and 2016: a systematic review. J Infect. (2018) 76:522–8. 10.1016/j.jinf.2018.02.008 29477803

[23] Ma X Yang M. The correlation between High-risk HPV infection and precancerous lesions and cervical cancer. Am J Transl Res. (2021) 13:10830–6. 34650762 PMC8507010

[24] Kim JJ Simms KT Killen J Smith MA Burger EA Sy S. Human papillomavirus vaccination for adults aged 30 to 45 years in the United States: a cost-effectiveness analysis. PLoS Med. (2021) 18:e1003534. 10.1371/journal.pmed.1003534 33705382 PMC7951902

[25] Rujumba J Akugizibwe M Basta NE Banura C. Why don't adolescent girls in a rural Uganda District initiate or complete routine 2-dose HPV vaccine series: perspectives of adolescent girls, their caregivers, healthcare workers, community health workers and teachers. Public Libr Sci One. (2021) 16:e0253735. 10.1371/journal.pone.0253735 34185805 PMC8241119

[26] Altobelli E Rapacchietta L Profeta VF Fagnano R. HPV-vaccination and cancer cervical screening in 53 WHO European countries: an update on prevention programs according to income level. Cancer Med. (2019) 8:2524–34. 10.1002/cam4.2048 30993902 PMC6536990

[27] Luo JF Jin H Li BY Zhao NQ. Application of Restricted Cubic Spline in Nonlinear Regression. Chin J Health Stat. (2010) 27:229–32. 10.3969/j.issn.1002-3674.2010.03.00225637058

[28] Xu Y Tu LW Xu YJ Fu HN. Analysis on the Cognition and Influencing Factors of HPV Vaccination among Female College Students-Based on Health Belief Model. J Nurs Rehabi. (2020) 19:18–21. 10.3969/j.issn.1671-9875.2020.01.004

[29] Schaefer Ziemer K Hoffman MA. Beliefs and attitudes regarding human papillomavirus vaccination among college-age women. Health Psychol. (2013) 18:1360–70. 10.1177/1359105312462432 23188917

[30] Hopfer S. Effects of a Narrative HPV vaccination intervention aimed at reaching college women: a randomized controlled trial. Prev Sci. (2012) 13:173–82. 10.1007/s11121-011-0254-1 21993613

[31] Christy SM Winger JG. Mosher CE. Does self-efficacy mediate the relationships between social-cognitive factors and intentions to receive HPV vaccination among young women? Clin Nurs Res. (2019) 28:708–25. 10.1177/1054773817741590 29134823 PMC6103913

[32] Haehnel Q Whitehead C Broadbent E Hanson CL Crandall A. What makes families healthy? Examining correlates of family health in a nationally representative sample of adults in the United States. J Family Issues. (2022) 43:3103–26. 10.1177/0192513X211042841

[33] Hopfer S Clippard JR. College Women's HPV Vaccine Decision Narratives. Qual Health Res. (2011) 21:262–77. 10.1177/1049732310383868 20841433

[34] Schor E Starfield B Stidley C Hankin J. Family Health. Utilization and effects of family membership. Med Care. (1987) 25:616–26. 10.1097/00005650-198707000-00005 3695666

[35] Stout ME Christy SM Winger JG Vadaparampil ST Mosher CE. Self-efficacy and HPV Vaccine attitudes mediate the relationship between social norms and intentions to receive the HPV vaccine among college students. J Community Health. (2020) 45:1187–95. 10.1007/s10900-020-00837-5 32418009 PMC7606315

[36] Thompson EL Vamos CA Straub DM Sappenfield WM Daley EM. Human Papillomavirus Vaccine Information, Motivation, and Behavioral Skills among Young Adult US Women. J Health Psychol. (2018) 23:1832–41. 10.1177/1359105316672924 28810358

[37] World Health Organization. Cervical Cancer. Available online:https://www.who.int/news-room/fact-sheets/det-ail/cervical-cancer (accessed on February22, 2022).

[38] Chan PS Fang Y Chidgey A. Fong F, Ip M, Wang Z. Would Chinese Men Who Have Sex With Men Take Up Human Papillomavirus (HPV) Screening as an Alternative Prevention Strategy to HPV Vaccination? Front Med (Lausanne). (2022) 9:904873. 10.3389/fmed.2022.904873 35721088 PMC9205561

