# Corrigendum: A national cross-sectional study on the influencing factors of low HPV vaccination coverage in mainland China

**DOI:** 10.3389/fpubh.2023.1328661

**Published:** 2023-12-12

**Authors:** Xiangju Yin, Mengrui Zhang, Fei Wang, Yue Huang, Yuyao Niu, Pu Ge, Wenli Yu, Yibo Wu

**Affiliations:** ^1^School of Emergency Management, Henan Polytechnic University, Jiaozuo, China; ^2^School of Resource and Environment, Henan Polytechnic University, Jiaozuo, China; ^3^State Key Laboratory of Cognitive Neuroscience and Learning, Beijing Normal University, Beijing, China; ^4^Department of English, Faculty of Arts and Humanities, University of Macau, Macau, China; ^5^School of Foreign Languages, Weifang University of Science and Technology, Weifang, China; ^6^School of Public Health, Peking University, Beijing, China

**Keywords:** self-efficacy, family health literacy, restricted cubic spline, cervical cancer, HPV vaccination

In the published article, there were errors in Tables 1, 2 as published. In Table 1, the headings of columns 3 was displayed as “HPV vaccinated group” and the headings of columns 4 was displayed as “Non HPV vaccinated group.” The correct statement of column 3 is “Non HPV vaccinated group,” and the correct statement of column 4 is “HPV vaccinated group.” In Table 2, the third line of the age variable assignment was displayed as “ ≤ 18 = 0, 19–40 = 1, 19–40 = 2, ≥66 = 3.” The correct statement is “ ≤ 18 = 0, 19–40 = 1, 41–65 = 2, ≥66 = 3.” The corrected tables appear below.

**Table 1:** Comparison of differences between HPV vaccinated and unvaccinated groups.

**Indicators**	**Group**	**Non HPV vaccinated group**	**HPV vaccinated group**	***x*^2^ or *t***	***P***
Place of residence	Urban	3,309 (76.24%)	1, 031 (23.76%)	32.176	<0.001
	Rural	1,345 (83.08%)	274 (16.92%)		
Age (years)	≤18	546 (87.64%)	77 (12.36%)	112.734	<0.001
	19-40	2,192 (72.92%)	814 (27.08%)		
	41-65	1,544 (81.01%)	362 (18.99%)		
	≥ 66	372 (87.74%)	52 (12.26%)		
Highest education levels	Junior high school and below	1,177 (85.41%)	201 (14.59%)	68.495	<0.001
	Secondary and High School	790 (79.96%)	198 (20.04%)		
	College	562 (74.04%)	197 (25.96%)		
	Undergraduate	2,125 (74.98%)	709 (25.02%)		
Marital Status	Unmarried	1,924 (79.27 %)	503 (20.73 %)	3.302	0.069
	Married	2,730 (77.29 %)	802 (22.71 %)		
Type of medical insurance	Medical insurance or public funding	3,625 (77.94 %)	1,026 (22.06 %)	0.318	0.573
	Self-financed	1,029 (78.67 %)	279 (21.33 %)		
Capita household income/month (yuan)	≤3,000	1,579 (83.28 %)	317 (16.72 %)	74.23	<0.001
	3,001–6,000	1,806 (78.42 %)	497 (21.58 %)		
	6,001–9,000	717 (74.53 %)	245 (25.47 %)		
	≥9,001	552 (69.17 %)	246 (30.83 %)		
Scale	NGSES	28.69 ± 5.061	29.06 ± 5.631	−2.162	0.031
	PSSS	61 ± 12.284	61.07 ± 13.342	−0.152	0.879
	FHS-SF	38.58 ± 6.420	37.86 ± 6.763	3.432	0.001
	HLS-SF12	36.67 ± 5.722	37.28 ± 6.264	−3.156	0.002

**Table 2:** Variables and dummy variables assignment table.

**Variable name**	**Assignment description**
HPV vaccination	No = 0, Yes = 1
Place of residence	Rural = 0, Urban = 1
Age	* ≤* 18 = 0, 19–40 = 1, 41–65 = 2, ≥66 = 3
Capita household income/month (yuan)	* ≤* 3,000 = 0, 3,001–6,000 = 1, 6,001–9,000 = 2, ≥9,001 = 3
Highest education level	Junior high school and below = 0, junior college and high school = 1, college = 2, university undergraduate and above = 3
HLS-SF12, FHS-SF, NGSES	Continuous variables

The authors apologize for this error and state that this does not change the scientific conclusions of the article in any way. The original article has been updated.

